# No distinction in the gut microbiota between diarrhea predominant-irritable bowel syndrome and healthy subjects: matched case–control study in Thailand

**DOI:** 10.1186/s13099-021-00406-8

**Published:** 2021-03-03

**Authors:** Sawangpong Jandee, Suppana Chuensakul, Suppasil Maneerat

**Affiliations:** 1 Gastroenterology and Hepatology Unit, Division of Internal Medicine, Faculty of Medicine, Prince of Songkla University, Hat Yai, Songkhla, Thailand; 2 Division of Gastroenterology and Hepatology, Department of Internal Medicine, Faculty of Medicine, Naresuan University, Phitsanulok, Thailand; 3 Biotechnology for Bioresource Utilization Laboratory, Department of Industrial Biotechnology, Faculty of Agro-Industry, Prince of Songkla University, Hat Yai, Songkhla, Thailand

**Keywords:** Diarrhea, Irritable bowel syndrome, Microbiota, Probiotic

## Abstract

**Background:**

Alteration in the gut microbiota has been proposed in irritable bowel syndrome (IBS) pathogenesis, especially in the diarrheal type (IBS-D). We conducted this study to evaluate the fecal microbiota in Thai IBS-D patients when compared with healthy subjects as well as to evaluate the effects of probiotics on changes in the gut microbiota correlated with symptoms.

**Methods:**

A matched case–control study was conducted on diagnosed IBS-D patients, based on the Rome IV criteria and healthy controls. Stool samples were collected in preservation tubes. Bacterial deoxyribonucleic acid extraction was performed and amplified. Next, 16S ribosomal ribonucleic acid genes sequencing was performed to identify the microbiome in both the groups. IBS-D patients were provided with a probiotic mixture that was rich in *Lactobacillus acidophillus* and *Bifidobacterium bifidum* over 8 weeks. Changes in the symptoms, stool characteristics, and fecal microbiota were evaluated and compared with the corresponding baseline values.

**Results:**

Twenty IBS-D patients with 20 age and gender-matched controls were included in this study. The baseline characteristics were not significantly different between the groups, including the mode of birth and the history of breastfeeding in infancy. No significant difference was noted in the fecal microbiota between the IBS-D patients and controls. The IBS symptom severity scales (IBS-SSS) were not statistically different after probiotic prescription; although, the bowel movements, the sense of urgency to go to the toilet and passing of mucous stool had obviously decreased. No change was noted in the fecal microbiota after receiving the experimental probiotic, except for an increase in the proportion of *B. bifidum.*

**Conclusion:**

Alteration in the gut microbiota composition was probably not the main pathogenic mechanism in the Thai IBS-D patients assessed in this study. However, modifying microbiomes with potentially protective bacteria seems to be a beneficial therapy.

*Thai Clinical trial registry*: TCTR20191211006, Date of registration: 10 Dec 2019. Retrospectively registered, Clinical trial URL: www.clinicaltrials.in.th

## Introduction

Irritable bowel syndrome (IBS) is a common bowel disorder that result in significant impairment of the quality of life and high healthcare expense [1]. The prevalence of IBS has been reported to be 10–20% in the western communities [2], 6.5–10.1% in Asia, [3] and 4.4% in Thailand; which seems to be underestimated [4]. IBS is presently classified based on the ROME IV criteria and in accordance with the predominant bowel habits, as follows: IBS with constipation (IBS-C), IBS with diarrhea (IBS-D), IBS with mixed bowel habits (IBS-M), and unclassified IBS [5]. The symptoms included abdominal pain, bloating, flatulence, and abnormal bowel habits, which are common and have a significant impact on the daily life.

The disorder of the brain-gut axis has been accepted as the main concept of the IBS pathogenesis. The possible mechanisms identified for the gut-brain dysfunction include post infectious change, genetic factors, abnormality in serotonin metabolism, and disturbances in the intestinal microbiota related to low-grade inflammation and immune activation [6]. Several past studies have demonstrated that patients with IBS have alterations in the gut microbiota [7]. A real-time polymerase chain reaction (PCR) analysis from Finland exhibited lower amounts of *Lactobacillus *spp. in diarrhea predominant IBS patients [8]. In addition, a systematic review and meta-analysis conducted in 2017 revealed a downregulation of bacterial colonization, including that of the genus *Lactobacillus* and *Bifidobacterium*, particularly in IBS-D patients [9]. Furthermore, a past systematic review highlighted that probiotics is an effective intervention in IBS to improve the global symptoms and abdominal pain [10]. Nevertheless, most of the past studies in terms of gut microbiota diversity as well as the role of probiotics have been conducted in western countries. As a result, the data on gut microbiome in Southeast Asia countries, which differ from those of the western countries in terms of ethnicity, environment, and food culture, remain anecdotal.

Hence, in this study, we aimed to evaluate alterations in the fecal microbiota of Thai IBS-D patients relative to that in healthy subjects so as to confirm the main concept of IBS pathophysiology. In addition, we studied the effects of probiotics in cases of changes in the gut microbiota after adjuvant therapy, which correlated with the symptoms.

## Materials and methods

### Participants

Electronic search from the outpatient hospital database between January 2018 and March 2019 was initially applied to identify IBS-D patients in accordance with the international classification criteria of diseases 10 (ICD-10) diagnosis. A total of 20 IBS-D patients (aged 18–45 years) admitted to the Gastroenterology clinic, Songklanagarind Hospital and 20 healthy controls (age and gender-matched) were consecutively recruited in this study. IBS-D was diagnosed based on the Rome IV criteria. Patients who were pregnant, breastfeeding, suffering from severe systemic illnesses (such as liver cirrhosis, congestive heart failure, chronic renal failure, endocrine disorders, and malignancy), of known cases of human immunodeficiency virus infection, organs transplantation, and with a prescription history of probiotics, antibiotics, antipsychotic drugs, steroids, or immunosuppressive drugs within 3 months prior to the study were excluded. Written informed consents were obtained from all participants.

A past study by Malinen [8] demonstrated relative differences in 16S ribosomal ribonucleic acid (16S rRNA) copy number of *Lactobacillus *spp., which was significantly lower in IBS patients than in the control subjects (p = 0.019). We required at least 19 participants, per arm, to detect 90% power with alpha (α) = 0.05. The study was approved by the Office of Human Ethics Committee, Faculty of Medicine, Prince of Songkla University and registered at the Thai clinical trials registry.

### Patient involvement

The target participants, from initial electronic searching, were contacted and provided with an explanation concerning the research details, followed by requesting their participation; additionally, permission from their primary physicians was also obtained before initiating the informed consent process. Some questionnaires were applied, such as the Thai version, in order to be easily understood. The English version of the questionnaires were explained in detail by the same investigators before the patients made any decision. The patients could contact the investigators immediately if they had any problems related to the study. Once the trial had finished and completely analyzed, the participants were informed of the results by the investigators when they visited for their next follow-up examination.

### Study medication

We used Infloran^®^ as the probiotic in this study considering that the data from the systematic review and meta-analysis conducted in 2017 suggested a decrease in the colonization of *Lactobacillus* and *Bifidobacterium* genus in IBS-D patients [9]. Each 250 mg capsule of Infloran contained *Lactobacillus acidophilus* (109 CFU) and *Bifidobacterium bifidum* (109 CFU). The patients were instructed to keep their study medications refrigerated at 2–7 °C. The probiotics were transferred onto ice cooler bags during their transfer from the hospital to the patients’ home.

### Study protocol

**Fig. 1:**
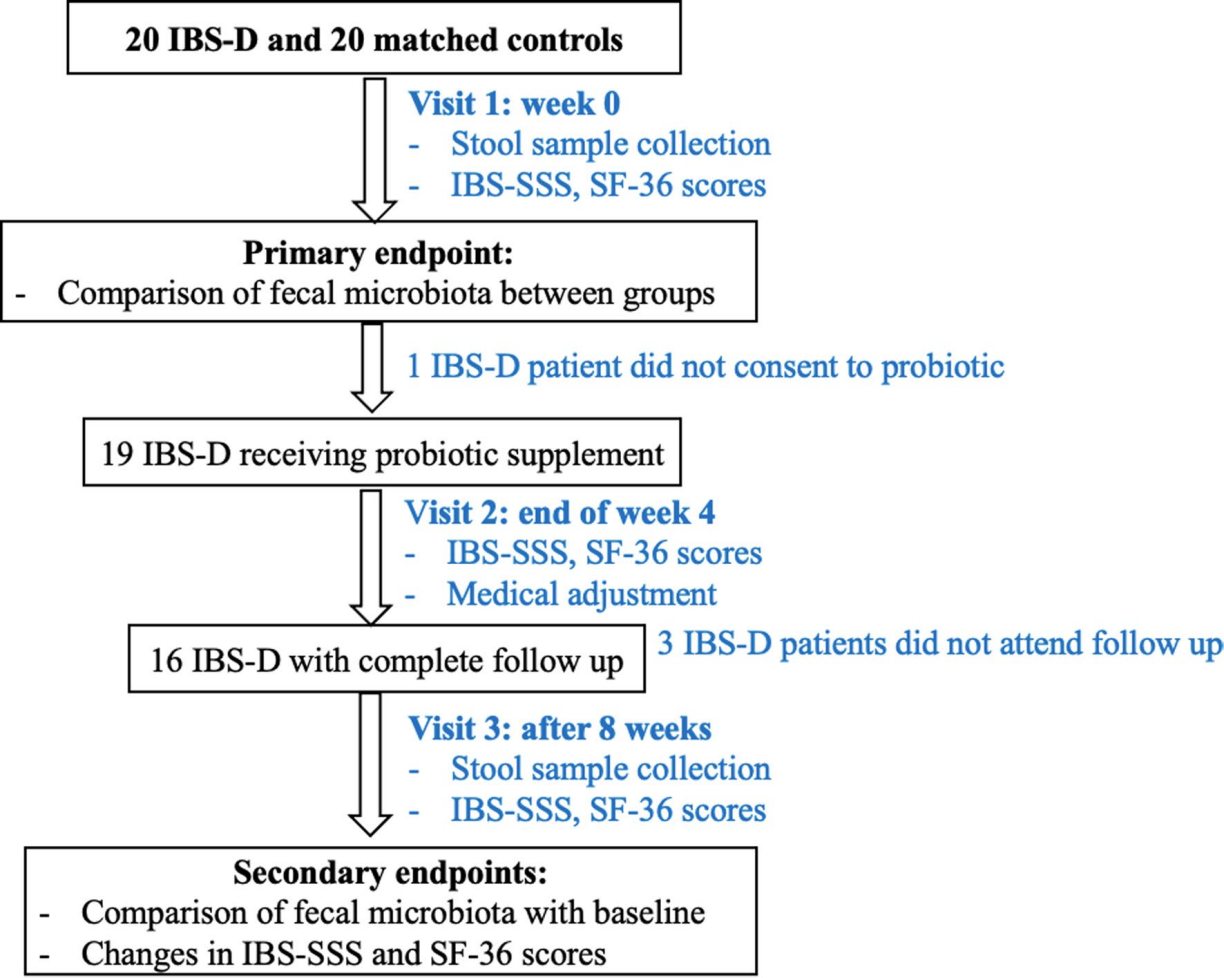
Flow diagram of study. *IBS-D* irritable bowel syndrome with diarrhea, *IBS-SSS* IBS symptom severity scale, *SF-36* 36-item short form health survey

This trial was a matched case–control study. First, stool samples from the IBS-D patients and healthy controls were collected. The demographic data, including gender, age, mode of birth, history of breastfeeding in infancy, and exercise habits were collected form the subjects. Only IBS-D patients were prescribed with the probiotic medication in doses of 1 capsule thrice a day (total 3 × 10^9^ lyophilized bacteria) over 8 weeks. The IBS symptom severity scale (IBS-SSS), bowel habit characteristics, and the 36-item short form health survey (SF-36) were evaluated at the baseline and after 4 and 8 weeks. At the end of week 4, patients were scheduled for evaluation and medical adjustment, if required. Patients who completed 8 weeks of a prescribed course of the probiotics adjuvant were requested to submit their stool samples again (Fig. 1).

### Clinical symptoms evaluation

IBS-SSS [11], a 5-domain instrument, was used to measure the extent of abdominal pain, bloating, satisfaction with bowel habits, and overall interference with the quality of life. The IBS-SSS total score ranged from 0 to 500, and a higher score indicated a worsened condition. Scores < 175 represented mild IBS, 175–300 represented moderate severity, and > 300 represented severe IBS. The IBS-SSS was evaluated by the physician at each visit. The SF-36 life quality scale, which provided psychometrically based physical component summary and mental component summary scores, consisted of the following: physical function, physical role difficulty, emotional role, liveliness, pain, mental function, social relationship, and perception of general health. The scores range between 0 and 100 points and were applied in the Thai version [12].

### Fecal sample collection

Fecal samples for bacterial analysis were collected in stool nucleic acid collection and preservation tubes (Norgen Biotek), which could stabilize the stool deoxyribonucleic acid (DNA) during the dispatch to the laboratory at the room temperature, warm (37 °C), or hot (50 °C) temperatures for up to 6 days. The stool samples were collected twice for each IBS-D patient (at the baseline and after prescribing the probiotics) and one-time for the healthy control; there was no restriction on the time of the day for collection and it was selected by the subject based on convenience. The participants were advised to scoop the stool sample from 3 sites using the spoon attached to the cap of the bottle and place the stool into the tube until the liquid reached the reference line, which is equivalent to 2 g of stool input.

### Amplification of bacterial genomic DNA by PCR

The total genomic bacterial DNA was extracted from the stool samples, as per the protocol of PureLink™ Genomic DNA Purification Kit (Invitrogen, Carlsbad, CA, USA). The total fecal bacteria were analyzed twice before amplifying the V6-V8 region for 16S rRNA gene [13, 14]. The reaction mixtures were subsequently cooled down to 4 °C. The size and amounts of PCR products were estimated by analyzing 5 µL of the samples on agarose gel electrophoresis using standard DNA markers. The amplified DNA was stored at − 20 °C until further use.

### Sequencing of 16SrRNA genes: real-time PCR and 2-step reverse transcription-PCR (RT-PCR)

We focused on the bacterial strains that seemed to be distinct between IBS-D and healthy subjects, particularly *Bifidobacterium* and *Lactobacillus*, as well as the common strains of gut bacteria, as suggested by previous studies. Oligonucleotides and optimized PCR conditions used in this study are summarized in Table 1. Primer specificity for 16S rRNA gene sequences were amplified from the total fecal DNA with the primers 27F and 1492R. The 16S rRNA gene sequences were used as templates to test the specificity of the target primers. The annealing temperature, which maximized primer specificity in vitro, was determined by using the targeted and non-targeted clones, which has been described above as templates; with the annealing temperatures of 55–60 °C. For PCR reactions, the SensiFAST Probe SYBR No-ROX (Bioline, Sydney, Australia) was applied to all groups of bacteria. PCR was performed as per the following steps and applied for all primers; the DNA templates were denatured at 95 °C for 10 min, followed by 40 cycles of denaturation at 95 °C for 30 s and annealing at 55–60 °C for 45 s. The pooled products were sequenced using the Rotor-Gene Q (QIAGEN’s real-time PCR cycler, Germany) melting-curve analysis performed after amplification. The cycle threshold values as well as the baseline settings were determined by using automatic analysis settings.

**Table 1:** Oligonucleotides and optimized PCR conditions of the pathogen panel applied in this study

PCR test (Amplicon Size, Mg^2+^, T_m_, detection)	Sequence (5′ → 3′)	References
*Bacteroides-Prevotella-Porphyromonas* (140 bp, 3 mM,55 °C, SYBR)	F:5′-GGTGTCGGCTTAAGTGCCAT-3′ R:5′-CGGA(C/T)GTAAGGGCCGTGC-3′	[15]
*Bifidobacterium *spp*.* (243 bp, 3 mM, 55 °C, SYBR)	F:5′-TCGCGTC(C/T)GGTGTGAAAG-3′ R:5′-CCACATCCAGC(A/G)TCCAC-3′	[15]
*Campylobacter *spp*.* (246 bp, 3 mM, 55 °C, SYBR)	F:5′-GGATGACACTTTTCGGAG-3′ R:5′-AATTCCATCTGCCTCTCC-3′	[15]
*C. difficile* (157 bp, 3 mM, 55 °C, SYBR)	F:5′-TTGAGCGATTTACTTCGGTAAAGA-3′ R:5′CCATCCTGTACTGGCTCACCT-3′	[15]
*C. perfringens* group (120 bp, 3 mM, 55 °C, SYBR)	F:5′-ATGCAAGTCGAGCGA(G/T)G-3′ R:5′-TATGCGGTATTAATCT(C/T)CCTTT-3′	[15]
*Enterococcus *spp*.* (144 bp, 3 mM, 55 °C, SYBR)	F:5′-CCCTTATTGTTAGTTGCCATCATT-3′ R:5′-ACTCGTTGTACTTCCCATTGT-3′	[15]
*Veillonella *spp*.* (343 bp, 3 mM, 55 °C, SYBR)	F:5′-A(C/T)CAACCTGCCCTTCAGA-3′ R:5′-CGTCCCGATTAACAGAGCTT-3′	[15]
*B. fragilis* (176 bp, 3 mM, 55 °C, SYBR)	F:5′-GAAAGCATTAAGTATTCCACCTG-3′ R:5′-CGGTGATTGCTCACTGACA-3′	[16]
*E. coli* subgroup (340 bp, 3 mM, 61 °C, SYBR)	F:5′-GTTAATACCTTTGCTCATTGA-3′ R:5′-ACCAGGGTATCTAATCCTGTT-3′	[16]
*Lactobacillus *spp. (341 bp, 2 mM, 55 °C, SYBR)	F:5′-AGCAGTAGGGAATCTTCCA-3′ R:5′-CACCGCTACACATGGAG-3′	[17]
*B. bifidum* (278 bp, 2 mM, 55 °C, SYBR)	F:5′-CCACATGATCGCATGTGATTG-3′ R:5′-CCGAAGGCTTGCTCCCAAA-3′	[18]
*B. longum* group (106 bp, 4 mM, 55 °C, SYBR)	F:5′-CAGTTGATCGCATGGTCTT-3′ R:5′-TACCCGTCGAAGCCAC-3′	[18]
*L. acidophillus* (575 bp, 55 °C, SYBR)	F:5′-CACTTCGGTGATGACGTTGG-3′ R: 5′-CGATGCAGTTCCTCGGTTAAGC-3′	[19]

*PCR* polymerase chain reaction, *C. difficile Clostridioides difficile*,* C. perfringens* Clostridium perfringens, *B. fragilis: Bacteroides fragilis*, *E. coli Escherichia coli*, *B. bifidum Bifidobacterium bifidum*, *B. longum Bifidobacterium longum*, *L. acidophilus Lactobacillus acidophilus*

### Statistical analysis

IBS-SSS and SF-36 instrument scores were expressed as means ± standard deviation (SD), and used the Student’s unpaired t-test to test the differences. Categorical data were presented as frequencies and percent and analyzed using the Chi-square test. Wilcoxon ranked sum test was used for non-parametric values. P < 0.05 indicated statistically significant value. Enumerations of bacterial counts were transformed to log_10_CFU/mL. General estimate equation (GEE) was applied to estimate change in the bacterial counts after probiotic prescription in the IBS-D group. All analyses were performed using the computer-based R program version 3.4.2.

## Result

Twenty IBS-D patients and 20 matched healthy control subjects were initially recruited in this study. Most participants were men (60%). No baseline characteristics were significantly different between the groups, including the mode of birth and the history of breastfeeding in infancy. We noticed more exercise duration in a week in the healthy controls relative to that in the IBS-D group, although it did not reach statistical significance (Table 2). The consistency of the stool sample at the baseline for the IBS-D patients was mostly classified as Bristol type-4 (smooth, soft sausage, or snake) and type 5 (soft blobs with clear-cut edge), which accounted for 63% and 31.6%, respectively; meanwhile, in the control group, the stool type was mostly Bristol type-3 (sausage shape with cracks on the surface) and type 4.

**Table 2:** Baseline characteristics of the IBS-D patients and healthy control subjects

Baseline characteristics	IBS-D (n = 20)	Healthy control (n = 20)	p value
Age (years)			
Mean (SD)	33.5 (7.7)	33.9 (7.5)	0.90
Gender			
Male (%) Female (%)	12 (60) 8 (40)	12 (60) 8 (40)	1.0
BMI (kg/m^2^)			
Mean (SD)	21.7 (4.2)	23.4 (3.2)	0.16
Duration of exercise (%)			
< 30 min/week ≥ 30 min/week	10 (50) 10 (50)	4 (20) 16 (80)	0.09
Mode of birth (%)			
Cesarean section Normal labor	4 (20) 16 (80)	5 (25) 15 (75)	1.0
History of breastfeeding in infancy (%)			
< 6 months ≥ 6 months Unknown	6 (30) 13 (65) 1 (5)	11 (55) 8 (45) 1 (5)	0.25

### Microbiota analysis

**Fig. 2:**
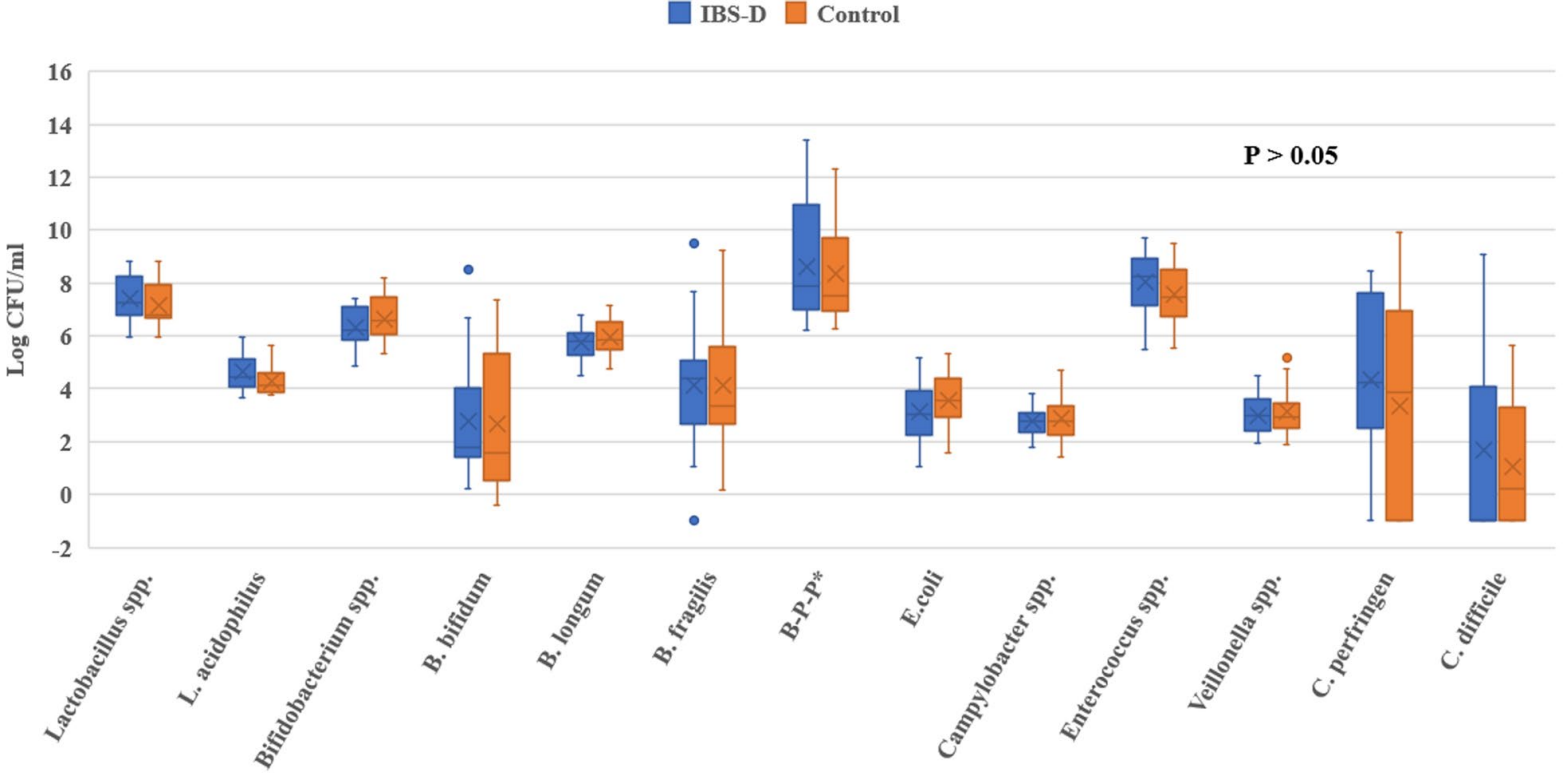
Comparison of 16s rRNA gene sequencing of fecal microbiota between the IBS-D patients and healthy control subjects. *IBS-D* irritable bowel syndrome with diarrhea, *L. acidophilus: Lactobacillus acidophilus, B. bifidum: Bifidobacterium bifidum, B. longum: Bifidobacterium longum, B. fragilis Bacteroides fragilis, B-P-P Bacteroides-Prevotella-Porphyromonas, E. coli Escherichia coli, C. perfringens* *Clostridium perfringens*, *C. difficile: Clostridioides difficile*

All real-time PCR reactions were performed in duplicate. The baseline fecal microbiota by RT-PCR was not statistically different between the IBS-D patients and healthy controls, including *Lactobacillus *spp. [7.3 (6.9, 8.2) vs. 6.8 (6.7, 7.8) log CFU/mL, p = 0.250] and *Bifidobacterium *spp. [6.2 (5.9, 6.9) vs. 6.6 (6.1, 7.4) log CFU/mL, p = 0.148], as mentioned elsewhere (Fig. 2).

### Severity scores, bowel habit, and SF-36 scores

From the 20 enrolled IBS-D patients, only 16 completed the following protocol for secondary endpoints (1 patient denied receiving probiotics, 3 patients did not attend their end of follow-up). The IBS-SSS scores, stool parameters, and SF-36 scores were assessed. At the baseline, most patients demonstrated a moderate severity of symptoms (95%), with only 1 patient (5%) showing mild symptoms. The IBS-SSS scores were not statistically different after receiving the probiotic. Although we noticed improvement in abdominal pain and in the number of days of experiencing pain at 4 weeks after probiotic prescription, the difference was not significant after 8 weeks. The maximum number of bowel movements in a day was significantly decreased after 4 weeks of receiving probiotics (p = 0.026), which continuously decreased through week 8 (p = 0.001). The percent of patients who passed mucous in their stool tended to decrease after 4 weeks of probiotic medication, to reach a significant difference at 8 weeks (57.9% vs 18.80%, p = 0.007), while those who had showed the symptom of hurrying to the toilet was significantly improved within the first 4 weeks (73.7% vs 37.50%, p = 0.015). The improvement of less body pain, which is a domain in SF-36, was evident after 4 weeks, although this effect was not noticed after week 8 (Table 3).

**Table 3:** Comparison of the IBS-SSS scores, stool parameters, and SF-36 scores at the baseline and after 4 and 8 weeks of probiotic prescription

	Baseline (mean ± SD)	End of week 4 (mean ± SD)	p value	End of week 8 (mean ± SD)	p value
Symptoms severity					
Total score	206.00 ± 70.60	176.20 ± 65.30	0.220	208.00 ± 77.80	0.920
Abdominal pain	32.10 ± 21.50	19.40 ± 24.90	0.018	30.60 ± 32.30	0.894
Number of days having pain	31.60 ± 30.80	10.00 ± 13.70	0.003	23.1 ± 33.4	0.472
Abdominal distension/tightness	36.30 ± 30.20	40.00 ± 33.70	0.260	36.90 ± 23.80	0.800
Satisfaction of bowel habits	50.50 ± 29.30	52.50 ± 26.20	0.870	59.70 ± 23.60	0.230
Affect or interfere with life	55.30 ± 25.00	54.40 ± 21.00	0.920	57.80 ± 27.30	0.800
Bowel habits					
Maximal number of bowel movements per day	3.47 ± 1.31	2.75 ± 1.18	0.026	2.44 ± 0.63	0.001
Passage of mucous	57.90%	31.20%	0.055	18.80%	0.007
Hurry/rush to the toilet	73.70%	37.50%	0.015	37.50%	0.019
SF-36					
Physical functioning	71.93 ± 24.88	78.64 ± 20.63	0.361	80.73 ± 27.67	0.245
Role-physical	69.74 ± 36.87	67.19 ± 44.46	0.860	71.87 ± 40.70	0.720
Bodily pain	42.11 ± 22.70	25.69 ± 16.56	0.003	30.55 ± 17.45	0.068
General health	44.13 ± 13.28	51.44 ± 22.59	0.220	40.38 ± 14.18	0.360
Vitality	65.35 ± 12.80	66.67 ± 15.21	0.830	63.54 ± 22.33	0.700
Social functioning	52.63 ± 18.47	60.71 ± 15.21	0.140	59.82 ± 9.36	0.200
Role-emotional	68.42 ± 34.20	68.75 ± 35.42	0.890	66.67 ± 43.88	0.780
Mental health	40.67 ± 22.95	42.61 ± 13.97	0.840	39.77 ± 20.96	0.870

*IBS-SSS* irritable bowel syndrome symptom severity score, *SF-36* 36-item short form health survey

### Adverse event

Every patient was encouraged to adhere to their probiotic prescription. Although no serious adverse events were reported, 2 patients required dose reduction after 4 weeks of usage due to worsening of abdominal bloating.

### Changes in the gut microbiota after probiotic prescription

**Fig. 3:**
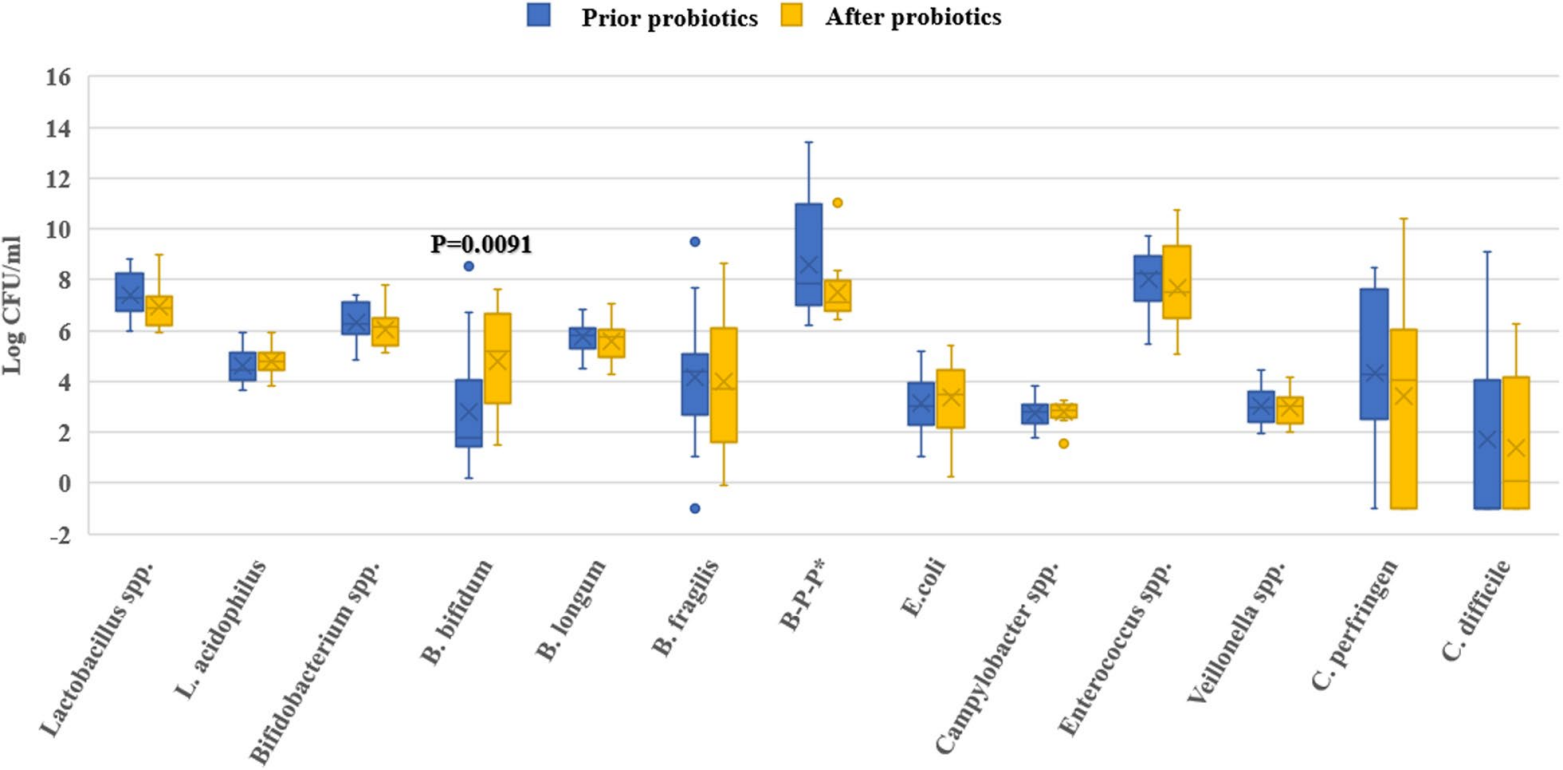
Comparison of 16s rRNA gene sequencing of fecal microbiota in IBS-D patients at the baseline and after probiotic prescription. *IBS-D* irritable bowel syndrome with diarrhea, *L. acidophilus Lactobacillus acidophilus, B. bifidum Bifidobacterium bifidum, B. longum Bifidobacterium longum, B. fragilis Bacteroides fragilis*,* B-P-P Bacteroides-Prevotella-Porphyromonas, E. coli Escherichia coli, C. perfringens* *Clostridium perfringens*, *C. difficile Clostridioides difficile*

After 8 weeks of probiotic prescription, which was rich in *Bifidobacterium* and *Lactobacillus,* fecal microbiota was analyzed and compared with that at the baseline to evaluate the impact of the probiotic consumption on the composition of the gut microbiomes. The results revealed a significant increase in the proportion of *B. bifidum* [2.8 (1.8, 3.8) vs. 4.8 (3.7, 5.9) CFU/mL, p = 0.009]; meanwhile, no significant changes were noted in the number of other gut flora (p > 0.05) (Fig. 3).

## Discussion

Disturbances to the intestinal microbiota have been proposed as possible mechanisms that result in the disorders of the brain-gut axis and IBS symptoms. The previous systematic review and meta-analysis of Liu et al. [9] revealed a downregulation of bacterial colonization, including that of the genus *Lactobacillus* and *Bifidobacterium,* in IBS-D patients. A recent systematic review of Pittayanon et al. [20] reported a decrease in the proportion of *Faecalibacterium* and *Bifidobacterium* in IBS patients, but increasing proportion of *Enterobacteriaceae, Factobacillaceae,* and *Bacteroides* relative to that in the control subjects. However, the results between the studies are lacking in consistency, due to their limitations; especially, considering the heterogeneity between the studies. Data of gut microbiota in Southeast Asia countries, which have different ethnicity, environment, and food culture from the West, remain anecdotal. This study is the first matched case control study conducted in Thailand and Southeast Asia to prove this hypothesis in IBS pathogenesis.

We demonstrated no distinction in the gut microbiota between the IBS-D patients and healthy controls in the Thai populations. Principally, host factors, such as genetics, age, and delivery pattern, and environmental factors, such as hygiene, diet, and antibiotics usage, can affect the gut microbiome [21]. In this study, we inclusively evaluated these mentioned factors and noted that even the mode of delivery, history of breastfeeding in infancy, and physical activity were not significantly different between the groups. These findings may be explainable for the non-distinction of gut microbiota between healthy and IBS-D patients. However, we did not evaluate the food diary for dietary patterns, which could have presented another important factor that influence the gut microbiome. In addition, the discordant results of gut microbiota between our study conducted in Thailand and those conducted in the western countries might be the effect of different dietary lifestyles, because of the types of consuming food can have a significant influence on the gut microbiota [22]. The western diet, which is high in fat and low in fiber, is related to a decrease in overall total bacteria and beneficial Bifidobacterium and Eubacterium species [23, 24]. Therefore, we may suppose that alteration of the gut microbiota is not the main pathogenic mechanism of IBS-D in Thai patients.

Theoretically, IBS is a gut-brain disorder with bothersome symptoms that can be precipitated from other explainable mechanisms apart from the alteration of the gut microbiota, including those of genetic factors, post infectious changes, low-grade mucosal inflammation, abnormalities in the serotonin metabolism, alteration in the brain functions, and mood disorders [6]. Although an approach based on the underlying pathophysiology can help develop a targeted therapy, it is difficult in clinical practice to accurately identify and measure the impact of individual underlying IBS mechanism.

Despite no differences in the gut microbiota between IBS-D patients and healthy controls, we noticed positive effects of probiotic use on abdominal pain, the number of days since having pain, and bodily pain domain in SF-36 after a short period of probiotic administration. Moreover, the number of bowel movements in a day, and the need to rush to the toilet was markedly decreased as of 4 weeks, while the symptoms of passing mucous stool reached statistical significance at 8 weeks. This observation demonstrated that modification of the gut microbiota by supplementing potentially protective bacteria act as an additional therapy for Thai IBS-D patients; particularly with probiotics containing *Bifidobacterium* and butyrate-producing bacteria. This could be because butyrate is an essential metabolite in the human colon. In addition, it is the preferred energy source for the colon epithelial cells, which contributes to the maintenance of the gut barrier functions and possesses immunomodulatory and anti-inflammatory properties [25]. In addition to the above, *Bifidobacterium longum* can decrease the 4-cresol sulfate levels after host bacterial interaction. This substance influences the dopamine/noradrenaline pathways and then reduces the depression scores and improves the quality of life [26].

Despite the lack of reports on serious adverse events after probiotic ingestion, 2 patients required dose reduction after 4 weeks due to worsening abdominal bloating. Based on our results, we suggested a 4–8-week duration as the appropriate period for probiotic prescription in Thai IBS-D patients so as to improve bodily pain, the frequency of bowel movements in a day, the need to rush to the toilet, and passing of mucous stool. This medication may also have some benefits with longer-term use if it is well tolerated by the patients.

After probiotic supplement, only *B. bifidum* significantly increased in count, while the others did not change statistically. Thus, it may be assumed that the positive results from good bacteria supplement arises from host bacterial interactions, rather than from any alteration in the gut flora.

Our study has several strengths. For instance, this was a matched case control design and included a specific subgroup of IBS-D patients, which minimized possible biases and confounders. We employed the standardized methods for stool collection, bacterial DNA extraction, amplification, and analysis. Importantly, this is the first study to provide gut microbiota information from Southeast Asia, which is different from those of the West, and facilitated the better understanding of the pathogenesis of IBS.

We also identified some limitations in the study. First, our results do not represent the whole microbiota diversity; however, we attempted to include most studied and cited bacterial studies from the IBS literature. Second, the study was not designed to calculate the sample size in order to test for the effects of the probiotic, which was the secondary objective. Moreover, it was a single-arm, open-label trial, which may present with possible bias from placebo effect, but it could not be responsible for the significant improvement of stool frequency and the passage of mucous stool in our study.

## Conclusion

According to our results, any alteration in the gut microbiota was probably not the main pathogenic mechanism among the Thai IBS-D patients. Modifying the microbiome strategies for IBS treatment outcomes in Southeast Asia populations may be different from those of the western countries. However, the use of a protective bacterial supplement seems to be a beneficial therapy, without altering the gut flora. Further study of a multicenter design are warranted to confirm our findings. Moreover, a double-blind, randomized controlled trial or a cross-over study may be useful to prove the efficacy of probiotics among Southeast Asia populations.

## Data Availability

All data generated or analyzed during this study are included in this published article.
